# Quantification of circulating steroids in individual zebrafish using stacking to achieve nanomolar detection limits with capillary electrophoresis and UV-visible absorbance detection

**DOI:** 10.1007/s00216-015-8785-0

**Published:** 2015-06-04

**Authors:** Vincent T. Nyakubaya, Brandon C. Durney, Marriah C. G. Ellington, Amber D. Kantes, Paige A. Reed, Shaylyn E. Walter, Jennifer Ripley Stueckle, Lisa A. Holland

**Affiliations:** C. Eugene Bennett Department of Chemistry, West Virginia University, Morgantown, WV 26506 USA; Department of Biology, West Virginia University, Morgantown, WV 26506 USA

**Keywords:** Bioanalytical methods, Bioassays, Biological samples, Capillary/electrophoresis, Endocrine disruptors

## Abstract

Capillary electrophoresis and UV-visible absorbance detection are used with sample stacking to achieve detection limits ranging from 0.2 to 2 ng/mL (0.8 to 6 nM) for steroids. Stacking is accomplished using negatively charged cyclodextrin steroid-carrier molecules at a discrete pH interface between the reconstituted sample and the separation electrolyte. Steroids are then separated in under 5 min using capillary electrophoresis that incorporates secondary equilibria via sodium dodecyl sulfate and cyclodextrin. The effectiveness of the method for measurements of multiple steroids in limited sample volumes is demonstrated in individual female fish with total circulating blood volumes of 5 μL or less. Steroid recoveries from plasma following a sample processing method developed with commercial extraction cartridges range from 81 to 109 % for 17α,20β-dihydroxy-pregn-4-en-3-one, testosterone, 11-ketotestosterone, estrone, 17β-estradiol, and 17α-ethinyl estradiol. When applied to reproductively active female zebrafish, changes were detected in the levels of circulating steroids as a result of exposure to different solvents and 17β-estradiol.

**Electronic supplementary material:**

The online version of this article (doi:10.1007/s00216-015-8785-0) contains supplementary material, which is available to authorized users.

**Graphical abstract:**
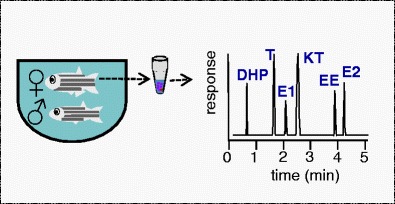
Steroids are measured in individual zebrafish subject to chemical exposure

## Introduction

Endocrine-disrupting chemicals mimic natural hormones and lead to impaired reproduction and adverse health outcomes [1]. Studies with zebrafish are integral to assessing the effects of endocrine-disrupting chemicals on human health because genes, development, and the hypothalamic-pituitary-gonadal axis are similar to those of humans [2]. Chemical biomarkers in the fish, such as steroid hormones and proteins, are measured to evaluate endocrine disruption [3]. Circulating steroids are effective to study both genomic and non-genomic mechanisms of action of the endocrine system, because it is well established that reproduction involves synchronized changes in steroidal hormones [4]. In female fish, changes in levels of 17α,20β-dihydroxy-pregn-4-en-3-one, testosterone, estrone, and 17β-estradiol induce vitellogenesis or ovulation. Endocrine-disrupting chemicals impact levels of multiple circulating steroidal hormones because they are regulated through interrelated and complex biological pathways. A set of steroid hormones must be monitored in an individual to elucidate the physiological response to toxicants because pooling plasma samples disproportionally normalizes the surges in steroid levels from outliers.

Regulated zebrafish exposure studies for endocrine disruption utilize fish that are 16 ± 2 weeks of age [5], even though the total blood volume at this age is 5 μL or less. Measurements of multiple circulating steroids with mass spectrometry or immunoassays are challenging in individual laboratory fish because of the limited blood volume and the number of animals investigated. Steroid hormones determined in plasma with mass spectrometry are coupled to gas chromatography or liquid chromatography [6–9]. The limited steroid volatility necessitates chemical derivatization for gas chromatography methods [6, 7]. The moderate steroid ionization efficiency of liquid chromatography generates high mass detection limits, which requires the use of plasma volumes greater than 5 μL [8]. Steroid analyses accomplished using immunoassays can be performed on small plasma volumes. Only a single antibody can be assayed for a single steroidal compound, and cross-reactivity towards other steroid-like compounds must be characterized. Additionally, antibodies are not commercially available for all steroids of interest, and the performance of each antibody must be validated for plasma samples to verify that antibody-antigen binding is not affected by interfering compounds in plasma for a particular species. Measurement of circulating steroids in zebrafish has been performed using immunoassays on pooled samples and is limited to determinations of estradiol [10–14], testosterone [10–14], or 11-ketotestosterone [14]. Immunoassay measurements of a single steroid in individual samples have been reported [15–17]. Estradiol, testosterone, and 11-ketotestosterone were measured in individual zebrafish aged 4–6 months that yielded plasma volumes up to 10 μL [18], while estradiol and testosterone were simultaneously measured in individual zebrafish older than 17 weeks [19, 20].

Direct detection of steroids is feasible with UV-visible absorbance detection, but detection limits achievable with capillary electrophoresis are in the micromolar range [21]. This problem is addressed by using different stacking methods to increase the amount of steroid loaded into the capillary without increasing the band broadening associated with large injection volumes [22, 23]. Analyte bands can be compressed based on differences in mobility in zones of background electrolyte of discontinuous conductivity as in field amplified stacking. Acidic or basic functional groups of an analyte can be harnessed by creating discontinuous regions of pH. For charged analytes, these techniques generate concentration factors up to 6000-fold [22]. Stacking neutral steroids is more difficult, but can be accomplished with carrier molecules, such as borate, sodium dodecyl micelles, or cyclodextrins, that form a steroid complex that can be stacked [21, 24–26]. A steroid detection limit of 118 ng/mL has been realized with UV-visible absorbance by creating large injection plugs of steroids solubilized in sulfated cyclodextrin carriers that migrate out of the injection zone and transfer steroids at the interface of the cholate micelles [21]. By filling the entire capillary with sample and sweeping the steroids into a smaller band prior to separation, steroid detection limits of 30 ng/mL were realized [24]. Large-volume sweeping generated a 5-ng/mL detection limit for testosterone by repeating the filling and micelle sweeping five times in a single capillary [25]. Stacking enhancement of steroids can be increased by combining multiple mechanisms for stacking. The combination of both a dynamic pH junction with sweeping, which resulted in 30-fold stacking enhancement for six steroids, was better than using only one mode of stacking [26]. UV-visible absorbance detection using pH-mediated sample stacking of the anionic stacking reagent carboxymethyl-β-cyclodextrin as a carrier for multiple steroids was optimized [27] and generated detection limits ranging from 0.8 to 4 ng/mL [28, 29].

In the current study, the pH-mediated stacking was combined with field amplified stacking to reduce the detection limits to 0.2 to 2 ng/mL (0.8 to 6 nM) for six natural and synthetic steroids. The method of processing the plasma was modified to generate recoveries ranging from 81 to 109 % from 5-μL plasma volumes. With these changes, the method was suitable to detect and quantify 17α,20β-dihydroxy-pregn-4-en-3-one, testosterone, 11-ketotestosterone, estrone, 17β-estradiol, and 17α-ethinyl estradiol in 5-μL plasma samples. The role and effects of circulating estrogenic steroids in females are well documented in fish that produce larger plasma volumes [30, 31], but not in small model female fish. The applicability of the method was tested by measuring circulating steroids following exposure of individual female zebrafish to 17β-estradiol, which is a positive control for estrogenic activity [5]. This new analytical technology provided unprecedented information about the effects of 17β-estradiol as well as the delivery solvent.

## Experimental

### Sample processing

Additional experimental details are provided as electronic supplementary material. Processing and sample analysis are briefly summarized here. Plasma samples were diluted with deionized water to bring each sample up to a volume of 25 μL, mixed, and then extracted in 75 μL of ethyl acetate. The extraction was repeated three times, and each ethyl acetate extraction combined to a total volume of 225 μL. The pooled ethyl acetate was evaporated to dryness at ambient temperature using a SpeedVac concentrator with a refrigerated vapor trap (Thermo Scientific, Waltham, MA, USA) in approximately 15 min. Once dried, the samples were reconstituted in a 200-μL solution of 1:3 water–1 % formic acid in acetonitrile. This 200-μL solution was then applied to a Hybrid SPE phospholipid cartridge (Sigma-Aldrich, St. Louis, MO, USA), which was then rinsed with an additional 200 μL solution of 1:3 water–1 % formic acid in acetonitrile. A total volume of 400 μL of 1:3 water–1 % formic acid in acetonitrile was collected and evaporated to dryness at ambient temperature using a SpeedVac concentrator in approximately 90 min. The dried fraction was reconstituted in 1000 μL 90 % 5 mM 3-(*N*-morpholino)-propanesulfonic acid and 10 % methanol and applied to a Discovery reversed-phase cartridge (Sigma-Aldrich) that had been conditioned with 5 mL of methanol followed by 5 mL of deionized water. The reversed-phase cartridge was then washed with 2 mL of deionized water. After this wash, the steroids were eluted with a 0.5 mL volume of methanol, which was collected and then evaporated to dryness at ambient temperature using a SpeedVac concentrator in approximately 60 min. The dried sample was reconstituted in 30 μL of the stacking solution.

### Steroid separation

The neutral steroidal compounds are separated based on secondary equilibria with sodium dodecyl micelles and hydroxypropyl β-cyclodextrin. The separation is accomplished with reversed polarity in under 5 min using an acidic background electrolyte to suppress electroosmotic flow. The charged steroid-cyclodextrin complex is injected electrokinetically into the separation capillary but becomes neutral when it encounters the acidic pH of the separation buffer in the capillary. During injection, the migration velocity of the neutral complex drops to zero, stacking analyte within the capillary prior to separation. Capillary electrophoresis separations were accomplished at 25 °C using a 25 μm id, 360 μm od, 30-cm-long fused silica capillary (Polymicro Technologies, LLC, Phoenix, AZ, USA) with an effective length of 10.2 cm and a background electrolyte comprised of 30 mM sodium dodecyl sulfate, 13 mM hydroxypropyl-β-CD, and 200 mM phosphate buffered at pH 2.5. Separations were performed at 16 kV with reversed polarity. The smaller inner diameter capillary is used to maintain currents at or below 35 μA. The sample was introduced using a 10-kV, 60-s pH-mediated electrokinetic stacking accomplished by reconstituting standards or samples in 30 μL of stacking electrolyte comprised of 1 mM carboxymethyl-β-cyclodextrin, 5 % methanol, and 5 mM CAPS buffered at pH 10.

## Safety considerations

Steroids and endocrine disruptors require the use of personnel protective equipment. Consult the MSDS for each compound prior to use.

## Results and discussion

### Improved stacking

The pH-mediated carboxymethyl-β-cyclodextrin stacking was most effective when the negatively charged cyclodextrins were driven from the sample vial into the separation capillary at the highest velocity achievable. This ensured that the maximum number of carboxymethyl β-cyclodextrin carriers accumulated at the interface of the basic stacking buffer and acidic separation buffer. A low-conductivity stacking solution induced a high velocity of the carboxymethyl-β-cyclodextrin ions, because these ions experienced a high electric field when a voltage was applied [32]. The solution conductivity, *σ*, is a function of the Faraday constant, *F*, the concentration, *C*, the electrophoretic mobility, *μ*, and the charge, *z*, as defined by Eq. 1 [33].

1 $$ \sigma =F\varSigma {C}_{\mathrm{i}}{\mu}_{\mathrm{i}}{z}_{\mathrm{i}} $$

**Fig. 1:**
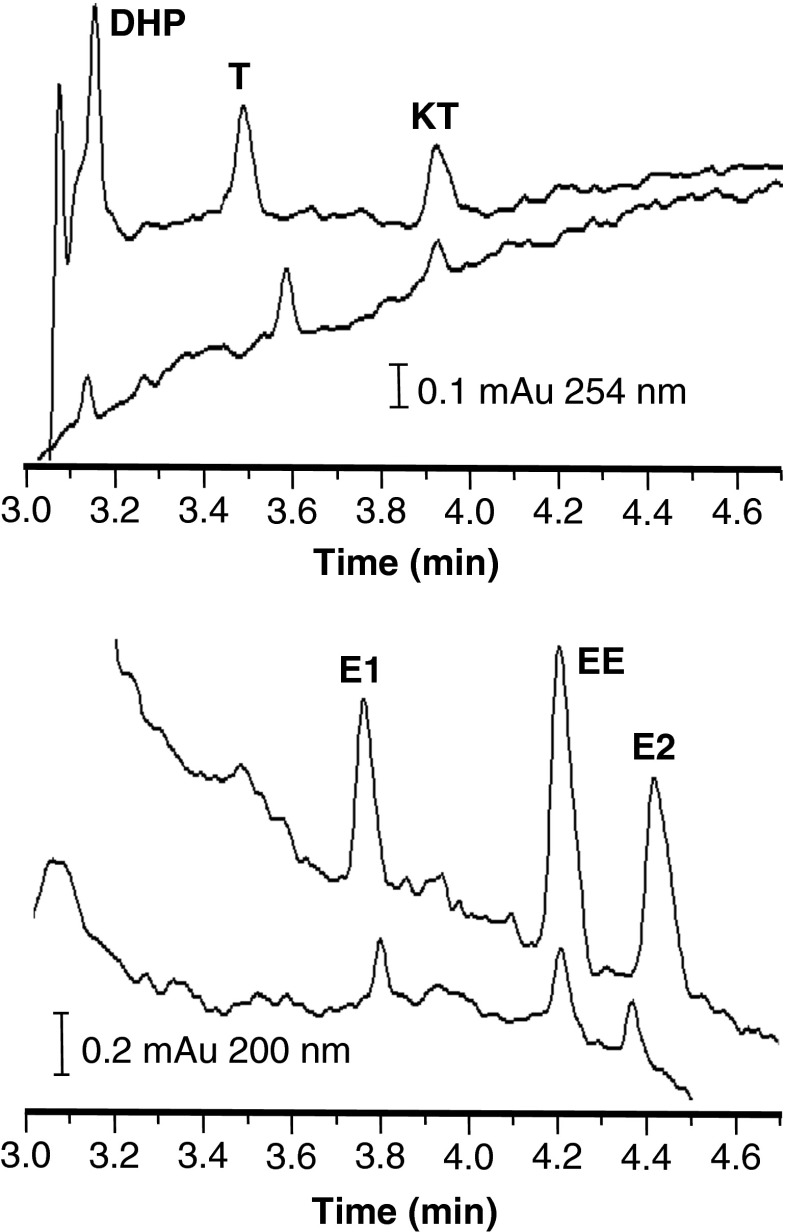
Electropherograms of steroid stacking at 200 and 254 nm demonstrate enhancement in area ranging from 3.7 ± 0.2 to 5.6 ± 0.3 observed with change in CAPS concentration from 50 to 5 mM CAPS. Peak labels are as follows: estrone (*E1*), 17α-ethinyl estradiol (*EE*), 17β-estradiol (*E2*), 17α,20β-dihydroxy-pregn-4-en-3-one (*DHP*), testosterone (*T*), and 11-ketotestosterone (*KT*). Separation conditions are described in the text

Previously, steroid analysis was reported using a stacking solution of 50 mM CAPS buffered to pH 10 [28, 29]. A decrease in the CAPS concentration in the stacking solution from 50 to 5 mM reduced the calculated conductivity of the sample matrix by a factor of eight. The steroidal compounds with high affinity for carboxymethyl-β-cyclodextrin should approach this concentration factor. As shown in Fig. 1, a stacking solution comprised of 1 mM carboxymethyl-β-cyclodextrin and 5 mM CAPS produced peak areas that were four to six times larger than those obtained with a stacking solution comprised of 1 mM carboxymethyl-β-cyclodextrin and 50 mM CAPS (see Electronic Supplementary Material (ESM) Table S1). The limits of detection determined with a 5-mM CAPS stacking buffer using standards ranging from 20 to 100 nM (*n* = 10) improved as follows: 17α,20β-dihydroxy-pregn-4-en-3-one (3.17 ± 0.06 nM), testosterone (2.8 ± 0.1 nM), 11-ketotestosterone (6.4 ± 0.2 nM), estrone (2.69 ± 0.09 nM), 17β-estradiol (0.79 ± 0.05 nM), and 17α-ethinyl estradiol (0.96 ± 0.04 nM). These detection limits, summarized in Table 1, were four to five times lower than that reported previously for 17α,20β-dihydroxy-pregn-4-en-3-one, 17β-estradiol, and 17α-ethinyl estradiol. CAPS concentrations less than 5 mM were evaluated; however, the stacking was not reproducible.

**Table 1:** Detection limits with improved steroid stacking

		17α,20β-Dihydroxy-pregn-4-en-3-one	Testosterone	11-Keto testosterone	Estrone	17β-Estradiol	17α-Ethinyl estradiol
5 mM CAPS (this work)							
LOD^a^	nM	3.17 ± 0.06	2.8 ± 0.1	6.4 ± 0.2	2.69 ± 0.09	0.79 ± 0.05	0.96 ± 0.04
	ng/mL	1.05 ± 0.02	0.81 ± 0.03	1.94 ± 0.06	0.73 ± 0.02	0.21 ± 0.01	0.28 ± 0.01
50 mM CAPS (refs [28, 29])							
LOD	nM	11 ± 3	4 ± 1	14 ± 3	3.1 ± 0.8	2.9 ± 0.06	4.7 ± 0.04
	ng/mL	4 ± 1	1.0 ± 0.3	4.2 ± 0.9	0.8 ± 0.2	0.79 ± 0.02	1.4 ± 0.02

Detection limits are obtained using a separation buffer comprised of 30 mM sodium dodecyl sulfate and 13 mM hydroxypropyl-β-CD in 200 mM phosphate buffered at pH 2.5. Injection conditions, separation voltage, and capillary dimensions are outlined in the text

^a^ *S*/*N* determined at a concentration of 100 nM for 17α,20β-dihydroxy-pregn-4-en-3-one (*n* = 10), 40 nM for testosterone (*n* = 10), 100 nM for 11-ketotestosterone (*n* = 10), 20 nM for 17β-estradiol (*n* = 10), 100 nM for estrone (*n* = 10), and 20 nM for 17α-ethinyl estradiol (*n* = 10)

### Method characterization

Analytical figures of merit of the improved stacking and separation method that were characterized using standards included the linear range of quantification, precision in migration time, and area for the new stacking conditions (see Table 2). The within-day and day-to-day variations in migration time precision were less than or equal to 1 and 10 % RSD, respectively. The higher variation in migration time across days did not impact quantification as calibration curves were constructed daily and repeated after ten consecutive runs. Nevertheless, the migration time variation across day may originate from a slight change in the separation background electrolyte, which was made daily. This was because the apparent mobility of steroidal compounds is based on competitive equilibria between anionic sodium dodecyl sulfate micelles and neutral hydroxypropyl β-cyclodextrin. Even though plasma samples were processed to facilitate stacking, each reconstituted plasma sample may contain compounds such as lipids and amphipathic molecules that hold potential to interfere with the suppressed electroosmotic flow as well as the secondary equilibria important to both stacking and separation. The method of sample preparation must minimize these effects, yet be compatible with plasma volumes as low as 5 μL.

**Table 2:** Analytical figures of merit of steroid determination with stacking and separation

	17α,20β-Dihydroxy-pregn-4-en-3-one	Testosterone	11-Keto testosterone	Estrone	17β-Estradiol	Ethinyl estradiol
Linear range^a^ (nM)	20–200	10–200	20–200	20–200	5–200	5–200
Reproducibility^b^ within day (*n* = 10)						
Time (min)	3.77 ± 0.02	4.17 ± 0.04	4.98 ± 0.06	4.57 ± 0.06	5.88 ± 0.04	5.50 ± 0.05
Area (×10^3^)	0.53 ± 0.05	0.61 ± 0.05	0.22 ± 0.01	0.80 ± 0.07	2.1 ± 0.1	1.94 ± 0.09
Reproducibility^b^ day to day (*n* = 3)						
Time (min)	3.4 ± 0.3	3.8 ± 0.3	4.4 ± 0.4	4.1 ± 0.4	5.3 ± 0.6	4.9 ± 0.5
Area (×10^3^)	0.52 ± 0.02	0.60 ± 0.01	0.22 ± 0.02	0.81 ± 0.06	2.0 ± 0.2	2.0 ± 0.3
Recovery^c^						
Standard (%)	86 ± 4	104 ± 4	92 ± 1	90 ± 9	86 ± 2	100 ± 2
Zebrafish, heparin (%)	93 ± 1	83 ± 6	109 ± 1	108 ± 5	81 ± 8	109 ± 8
Zebrafish, water (%)	95 ± 3	85 ± 1	107 ± 1	101 ± 3	102 ± 5	98 ± 4

^a^Curves at 254 nm were determined simultaneously for 17α,20β-dihydroxy-pregn-4-en-3-one, testosterone, and 11-ketotestosterone with *R* ^2^ ≥ 0.995. Curves at 200 nm, with *R* ^2^ ≥ 0.995, were determined simultaneously for 17β-estradiol, estrone, and 17α-ethinyl estradiol

^b^Reproducibility determined using 50.0 nM standards

^c^Recovery for the standard is based on 50 nM steroids, and fish plasma samples are based on spiking plasma with 50 nM steroids with 2 μL of added solution comprised of 6.5 mg of low molecular weight heparin/mL deionized water or with 2 μL of added deionized water and were subject to the full preparation protocol outlined in the text

### Sample processing

Steroid determinations were based on liquid-liquid extractions and on solid-phase extractions with commercially available cartridges. The purpose of the liquid extraction was to separate steroidal compounds from proteins, whereas the solid-phase extractions isolated steroids from amphipathic compounds, such as fatty acids, or molecules with different hydrophobicity, such as cholesterol. In a previous report, 0.1 mL volumes of plasma were processed with liquid-liquid extraction, and successive treatment with solid-phase extraction based on cationic exchange and reversed phase [28, 29]. This rigorous treatment did not remove interfering compounds that systematically reduced the recovery of estradiol [28, 29]. In the present work, the sample was subjected to an extraction with ethyl acetate to separate proteins from hydrophobic steroidal compounds. The compounds captured in the organic phase were applied to a Hybrid SPE phospholipid cartridge, which was comprised of zirconia-coated silica designed to retain phospholipids and elute all other hydrophobic components. The eluted fraction was then applied to a reversed-phase cartridge to desalt the sample, which was then reconstituted in stacking buffer. Fractions eluted from each of the three processing steps were dried under vacuum at ambient temperature, for 15, 90, and 60 min, respectively. The total time to process and evaporate the solvents was ~3 h. When sample processing was performed at ambient temperatures and required more than 4 h, the recovery was decreased due to thermal and photodegradation. The current sample protocol was performed in parallel for up to six samples for a total processing time of ~3 h.

The recovery of steroidal compounds in plasma samples was achieved by combining plasma from two fish and then splitting the combined samples into two equal volumes. Endogenous steroids were measured in one fraction, while six steroidal compounds were spiked into the second fraction. Analysis of each fraction provided a means to account for the endogenous steroids in the plasma fraction spiked with steroid standards. The results, summarized in Table 2, demonstrated recoveries ranging from 81 to 109 %. The fish used for the recovery study were not reproductively active; thus, the endogenous steroids were below the quantification limit of this method (see ESM Fig. S1). The steroid separations obtained from the plasma samples migrated within 5 % RSD of standard migration time. This shift in migration time that occurred with processed plasma samples was most likely due to the introduction of hydrophobic compounds that associate with the micelles and the cyclodextrins, altering the secondary equilibrium of the separation. Therefore, after the sample was separated and quantified, it was spiked with steroid standards (i.e., 17α,20β-dihydroxy-pregn-4-en-3-one, testosterone, 11-ketotestosterone, estrone, 17β-estradiol, and ethinyl estradiol) to verify the peak identification based on migration time.

For these determinations, the samples were quantified from a single separation to increase the sample throughput. In addition, the 30-μL sample volumes are prone to evaporation, the steroids are subject to thermal and photodegradation, and the hydroxide ion is electrolytically generated in the cathodic reservoir during the 60-s injection. A single plasma sample was spiked with 1.5 pmol each of 17α,20β-dihydroxy-pregn-4-en-3-one, testosterone, 11-ketotestosterone, estrone, 17β-estradiol, and ethinyl estradiol. Following sample preparation and reconstitution in stacking buffer, the concentration of each steroid was 50 nM when 100 % recovery was achieved. A single sample was subjected to three analyses (see ESM Table S2). The relative error associated with each measurement when estimated from the calibration curve ranged from 2 to 7 %, whereas the relative error determined from the standard deviation associated with the replicate measurements ranged from 3 to 7 %. Although the error generated from replicate measurements was similar to that obtained from a single measurement with a calibration curve, estrone degraded into two peaks, which is attributed to chemical processes associated with injection. To avoid issues associated with repeated injections, the measurement error (see Tables 1, 2, 3, and 4) was estimated from the error associated with the calibration curve. Although evaporation effects will not be reduced, chemical processes can be minimized by using methods that are compatible with large-volume hydrodynamic injections if sweeping and stacking can be achieved for the steroid standards, detection limits of 5 ng/mL are sufficient [25], and high-throughput measurements are not required.

**Table 3:** Effect of 17β-estradiol exposure on circulating steroids (ng/mL plasma) of female zebrafish

Treatment	17α,20β-Dihydroxy-pregn-4-en-3-one	Testosterone	11-Keto testosterone	Estrone	17β-Estradiol
Set 1: 16.9 weeks of age for exposure experiments					
Ethanol 1	ND	ND	ND	ND	ND
Ethanol 2	ND	ND	ND	ND	ND
Ethanol 3	ND	ND	ND	ND	188 ± 1
Ethanol 4	ND	37 ± 8	ND	ND	ND
Ethanol 5	115 ± 3	ND	ND	ND	ND
Results if pooled	23	7			38
100 ng/L estradiol 6	ND	32 ± 6	ND	ND	ND
100 ng/L estradiol 7	ND	33 ± 6	ND	ND	ND
100 ng/L estradiol 8	ND	ND	ND	214 ± 6	ND
100 ng/L estradiol 9	ND	34 ± 8	ND	118 ± 4	ND
100 ng/L estradiol 10	ND	ND	ND	223 ± 1	ND
Results if pooled		20		111	
Set 2: 33.3 weeks of age for exposure experiments					
Ethanol 11	112 ± 3	ND	110 ± 2	ND	ND
Ethanol 12	ND	ND	88 ± 2	ND	ND
Ethanol 13	74 ± 3	ND	77 ± 2	ND	ND
Ethanol 14	82 ± 4	ND	ND	ND	ND
Ethanol 15	ND	ND	ND	ND	ND
Results if pooled	54		55		
100 ng/L estradiol 16	ND	26 ± 6	46 ± 6	ND	39 ± 4
100 ng/L estradiol 17	ND	41 ± 5	34 ± 6	53 ± 2	ND
100 ng/L estradiol 18	ND	ND	53 ± 7	127 ± 2	ND
100 ng/L estradiol 19	ND	23 ± 7	70 ± 7	115 ± 2	ND
100 ng/L estradiol 20	ND	27 ± 7	ND	120 ± 2	ND
Results if pooled		23	41	83	8

**Table 4:** Effect of acetone exposure on circulating steroids (ng/mL plasma) of female zebrafish

Treatment	17α,20β-Dihydroxy-pregn-4-en-3-one	Testosterone	11-Keto testosterone	Estrone	17β-Estradiol
Acetone 21	70 ± 2	ND	40 ± 2	ND	ND
Acetone 22	ND	ND	ND	63 ± 6	39 ± 1
Acetone 23	105 ± 2	ND	108 ± 2	98 ± 7	128 ± 1
Acetone 24	ND	ND	ND	112 ± 7	ND
Acetone 25	ND	ND	ND	201 ± 6	ND
Results if pooled	35		30	95	33

### Exposure to 17β-estradiol

The steroid 17β-estradiol is used as a positive control for estrogenic activity [5]. A total of 40 fish were exposed to solvent or to 17β-estradiol dissolved in solvent. A single set is composed of 10 fish (i.e., five male and five female) maintained in a tank set up as a flow-through system for chemical exposure as mandated by guidelines reported by the Organisation for Economic Co-operation and Development [5]. Two sets were exposed to ethanol. Two sets were exposed to 17β-estradiol dissolved in ethanol. From these four exposure tanks, a total of 20 individual female fish (i.e., five females/tank) were studied by monitoring circulating steroids with the capillary electrophoresis method described in this paper. Blood collected from each fish in set 2 was spiked with 1.5 pmol of the synthetic steroid ethinyl estradiol prior to processing to confirm the compatibility of the method with an internal standard. Egg production was monitored during the exposure experiments because impaired reproduction is a critical indicator of endocrine disruption. The results summarized in ESM Fig. S2 demonstrate a change in egg production and hatching rate in the presence of estradiol, but the information provides little insight into the underlying mechanism of endocrine disruption.

The data summarized in Table 3 reveal the changes in total circulating steroid hormone that occurred in female fish exposed to 17β-estradiol. An advantage to monitoring steroids from individual fish is that information about hormonal balance is lost when samples are combined and analyzed. To demonstrate this, the data in Table 3 indicates the results obtained if the data were pooled instead of measured individually. These analyses of multiple steroids in single fish established for the first time changes in estrone in small model fish associated with estrogenic activity. Literature studies of circulating steroids in zebrafish are limited to classical steroids: 17β-estradiol, testosterone, and 11-ketotestosterone; however, no changes were detected in these steroids using capillary electrophoresis. With the use of a method to identify multiple steroids simultaneously, altered steroid levels are rapidly identified in individuals.

**Fig. 2:**
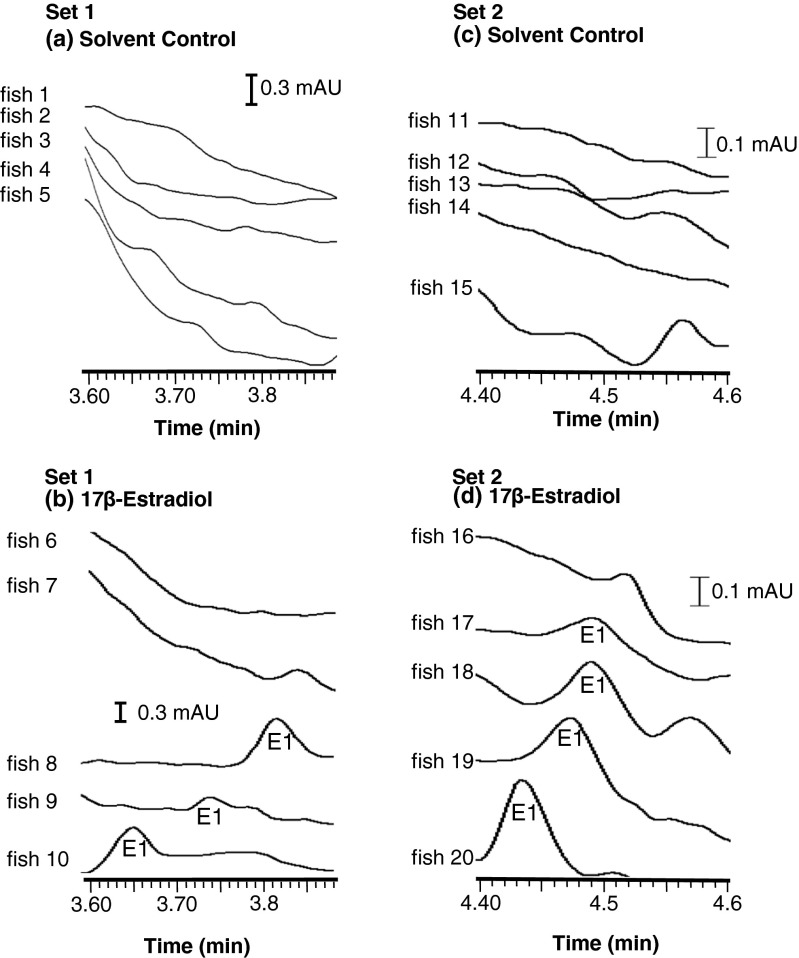
Stacked electropherograms from individual female zebrafish. *Set 1* fish are 16.9 weeks of age at the time of exposure to (**a**) ethanol solvent only or (**b**) 17β estradiol dissolved in ethanol solvent. *Set 2* fish are 33.3 weeks of age at the time of exposure to (**c**) ethanol solvent only or (**d**) 17β estradiol dissolved in ethanol solvent. Blood collected from fish analyzed in set 2 was spiked with ethinyl estradiol prior to sample processing to confirm the use of internal standards. Estrone is labeled as *E1*. Separation conditions are described in the text. Estrone is confirmed by spiking and then separating each sample after it was quantified

**Fig. 3:**
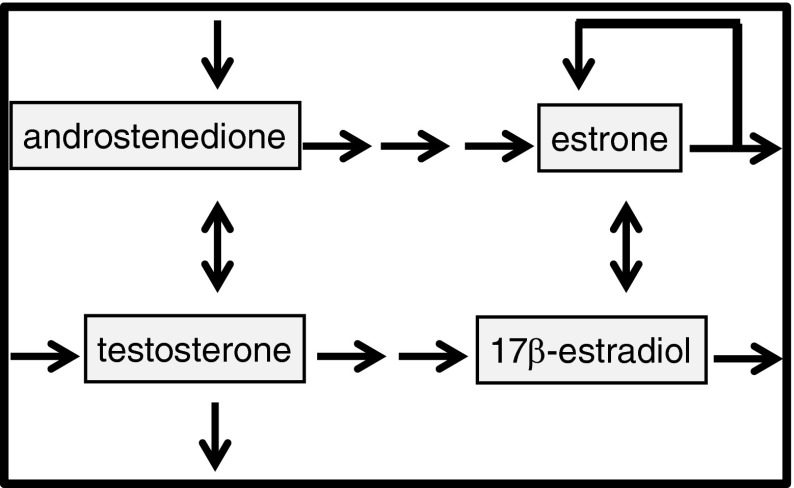
Interrelated pathways of testosterone, 17β-estradiol, and estrone biosynthesis

Relative to control fish, in which no estrone was detected, three fish in set 1, which was composed of fish that were 16.9 weeks old, had circulating estrone ranging from 118 to 233 ng/mL (see Fig. 2 and Table 3). Set 2 was composed of fish that were 33 weeks old, and following exposure to 17β-estradiol, four fish had circulating estrone ranging from 53 to 127 ng/mL (see Fig. 2 and Table 3). Thus, despite a difference in age, there is an increase in estrone levels in a majority of female fish following exposure to 17β-estradiol. Circulating estrone has never been detected in zebrafish but has been detected in larger fish, including flounder (*Platichthys flesus*) [6], yellow perch (*Perca fluviatilis*) [30], and catfish (*Clarias batrachus*) [31]. Based on another exposure study reported in the literature, estrone is implicated in early stages of vitellogenesis [34]. Estrone interacts with estrogen receptors and is more rapidly metabolized than synthetic steroids. Tools such as the Kyoto Encyclopedia of Genes and Genomes (KEGG) database demonstrate the interrelated pathways of steroid biosynthesis for zebrafish [35]. The scheme in Fig. 3, adapted from the KEGG database map, indicates that estrone is an endpoint of multiple synthesis pathways including 17β-estradiol via 17β-hydroxysteroid dehydrogenase.

### Effects of acetone on circulating estrone

In addition to ethanol [36], acetone [37] has also been reported as a solvent used to solubilize 17β-estradiol for exposure studies with zebrafish. The effect of acetone was assessed with reproductively active female zebrafish following a 7-day chemical exposure with a flow-through system. One set of 10 fish was exposed to acetone, and five individual female fish were studied using pH-mediated stacking and capillary electrophoresis. Estrone was detected in four female fish exposed to acetone with a circulating estrone ranging from 63 to 201 ng/mL (see Table 4). Different organic solvents may be used as the delivery vehicle for sparingly soluble compounds, although preliminary studies must be performed to verify that the presence of the delivery solvent has little or no effect on the endocrine system [38]. The generation of circulating estrone following acetone exposure warrants caution when using this solvent.

## Conclusions and future directions

The reported capillary electrophoresis method is amenable to the limited sample volume generated by individual zebrafish and provides a rapid means to measure multiple steroids in the large number of samples required for toxicity testing. The analysis of circulating steroids provided more insight about mechanisms of endocrine disruption relative to the information derived by monitoring physiological endpoints such as egg production. Estrone has not been previously detected in zebrafish due to the lack of an available validated antibody assay and because the blood volume is too low for analysis by mass spectrometry methods. An example of the advantage of this approach is supported by the measurement of circulating estrone. The method is currently being applied to study the effects of other endocrine-disrupting chemicals.

## Electronic supplementary material

ESM 1 (PDF 80 kb)
