# Supplementary material for: Quantification of circulating steroids in individual zebrafish using stacking to achieve nanomolar detection limits with capillary electrophoresis and UV-visible absorbance detection
Source: Anal Bioanal Chem. 2015 Jun 4;407(23):6985–93. doi: 10.1007/s00216-015-8785-0 (PMC4551537; doi:10.1007/s00216-015-8785-0)
Supplement: Supplementary file 1 — (PDF 80 kb) [file 216_2015_8785_MOESM1_ESM.pdf]

Analytical and Bioanalytical Chemistry

Electronic Supplementary Material

Quantification of circulating steroids in individual zebrafish using stacking to achieve nanomolar detection limits with capillary electrophoresis and UV-visible absorbance detection

Vincent T. Nyakubaya, Brandon C. Durney, Marriah C.G. Ellington, Amber D. Kantes, Paige A. Reed, Shaylyn E. Walter, Jennifer Ripley Stueckle, Lisa A. Holland

Abstract
This supporting information includes experimental details and summaries of the effects of concentration of the stacking buffer (Table S-1) and replicate injections (Table S-2). Figures demonstrating recovery with pooled fish plasma (Figure S-1A, S-1B) and egg production (Figure S-2) are also included.

Experimental

Reagents and materials. Acetonitrile, 3-[cyclohexylamino]-1-propanesulfonic acid (CAPS), carboxymethyl-β-cyclodextrin, 3-aminobenzoic acid ethyl ester, methanesulfonate salt (MS222), sodium dodecyl sulfate, and 11-ketotestosterone (CAS 564-35-2) were purchased from Sigma-Aldrich (St. Louis, MO, USA). Sodium phosphate monobasic salt was purchased from Fisher Scientific (Fair Lawn, NJ, USA). Steroids purchased from Steraloids, Inc (Newport, RI, USA) include, 17β-estradiol (CAS 50-28-2), estrone (CAS 53-16-7), 17α,20β-dihydroxy-pregn-4-en-3-one (CAS 1662-06-2), and 17α-ethinyl estradiol (CAS 57-63-6), and testosterone (CAS 58-22-0) was purchased from Cayman Chemical (Ann Arbor, MI, USA). Methanol was purchased from EMD Chemicals (San Diego, CA, USA). Ethyl acetate was purchased from Mallinckrodt (ACS grade, Phillipsburg, NJ, USA). The 3-(*N*-morpholino)-propanesulfonic acid (MOPs) was purchased from Alfa Aesar (Ward Hill, MA, USA) and hydroxypropyl-β-cyclodextrin was purchased from TCI (Portland, Oregon, USA) Deionized water was purified using ELGA Purelab Ultra (Lowell, MA, USA). Buffered solutions were prepared by weighing out solid reagents, dissolving them in deionized water, and titrating the solution to the desired pH with aqueous solutions of 1N hydrochloric acid or 1N sodium hydroxide, as appropriate. Stock solutions of each steroidal compound used for separation were prepared by weighing out the solid steroid reagent and dissolving it in methanol. Stock solutions of 17α-ethinyl estradiol used for toxicity experiments were prepared by weighing out the solid steroid reagent and dissolving it in ethanol. All stock were stored in a freezer at -20°C before use. Stock samples were diluted to a desired concentration each day prior to use with buffer containing 1 mM carboxymethyl-β-CD, 5% methanol, and 5 mM CAPS buffered at pH 10.

Fish maintenance. Animals were maintained as documented by the West Virginia University Animal Care and Use Committee Protocol 12-0101, Approved February 2012 or 14-1106 approved November 2014. Zebrafish housed at West Virginia University originated from Zebrafish International Resource Center (Eugene, OR). Fish were maintained in a balanced saline solution comprised of 17 mM NaCl, 0.66 mM $MgSO_4$, 0.40 mM KCl, 0.27 mM $CaCl_2$, 0.23 mM $NaHCO_3$. Tanks were treated with carbon filters and are maintained with 40% water changes every week. Algal growth was physically removed when visible. Water temperature was 25 to 27 °C under normal conditions. During breeding the temperature was 25 to 29 °C. Lights were set on a 14L:10D photoperiod. Fish were fed brine shrimp or flakes three times daily and were maintained at a density of 20 per 10 gallon tank.

Exposure experiments. Breeding fish were exposed for 7 days. Animals served as a control, or were exposed every day to solvent or 17β-estradiol. Exposure tanks containing male-female pairs were maintained on a 14 L:10 D light cycle and placed randomly in a water bath maintained at 25-29 °C for zebrafish. The 17β-estradiol stock solution was made in an ethanol solvent volume of 15 µL/L tank water. The ethanol solvent control was dosed at a concentration of 15 µL ethanol/L tank water. The acetone solvent was dosed at a concentration of 15 µL acetone/L tank water. Water renewal was accomplished with a flow-through system,

exchanging 25 liters of tank water throughout a 24 hour period. At the conclusion of the 1 week exposure, the female fish were sacrificed and blood was collected for analysis. Animals were euthanized with an overdose of MS222 (10 mg/100 mL aqueous). Blood was collected by cutting the isthmus above the heart and placing a 10 µL heparinized, disposable micropipet (Drummond Scientific, Fisher Scientific, St. Louis, MO) in the flow of arterial blood from the heart. Blood samples were transferred from the pipet to a 1.5 mL centrifuge tube (catalog #89000-028 VWR Radnor, PA) chilled in ice that contained 2 µL of deionized water or 2 µL of a solution of low molecular weight heparin dissolved in deionized water at a concentration of 6.5 mg heparin/ mL deionized water and then spun immediately in a 4 ºC centrifuge at ≥ 6,500 rpm for 10 minutes to separate the plasma. The plasma was transferred to a fresh centrifuge tube and the volume noted.

PROTOCOL: PLASMA PROCESSING

A) ethyl acetate extraction

- Add deionized water to the collected plasma already in a 1.5 mL centrifuge tube (catalog #89000-028 VWR) to bring the total volume up to 25 µL sample (the volume of water added is based on the recorded volume of the plasma). Mix the contents of the centrifuge vial with a vortex mixer for 10 seconds.
- Add 75 µL of ethyl acetate. Mix the contents for 1 minute with a vortex mixer. Centrifuge the vial for 5 seconds using the bench top picofuge. Collect the ethyl acetate phase (upper phase is ethyl acetate, lower phase is water). Do not remove water. It is better to leave residual ethyl acetate in the centrifuge tube than to remove all ethyl acetate and some water.
- The ethyl acetate extraction should be repeated two more times so that three extractions have been performed. Pool the ethyl acetate into a single 1.5 mL centrifuge vial.
- Dry the sample in the speed vac ~ 15 minutes

B) Hybrid SPE phospholipid cartridge (Sigma Aldrich) -The purpose of this step is to remove lipids from the plasma. Lipids solubilize the steroids and severely impede recovery of steroids with the reversed phase cartridge. This step can be multiplexed using the 8-port vacuum manifold.

- Reconstitute the samples in 200 µL of a solution that is 1:3 deionized water:1% formic acid in acetonitrile. Mix the contents of the 1.5 mL centrifuge vial using the vortex mixer for 1 minute.
- As per manufacturer protocol, do not rinse the phospholipid cartridges
- Load sample on the phospholipid cartridges and collect the eluted liquid. The lipids are retained on the cartridge, while the steroids are eluted. Rinse the phospholipid cartridges with an additional 200 µL of 1:3 deionized water:1% formic acid in acetonitrile. Collect the wash in the same vial that contains the steroid eluate.
- Dry the sample in the speed vac. This step takes about 90 minutes.

C) Discovery reversed phase cartridge (Sigma-Aldrich). The purpose of this step is to remove salts (first eluted wash fraction). This step can be multiplexed using the 8-port vacuum manifold.

- Turn on the vacuum to prepare the speed vac.
- Remove a fresh and clean high purity fraction of methanol in a secondary container (preferably 25 mL orange-capped bottle).
- Reconstitute samples in 200 µL of 25% methanol in freshly made **FILTERED** 5 mM MOPS buffer at pH 7. Mix the contents of the vial using the vortex mixer for 1 minute.
- Add 50 µL of methanol to the vial followed by 750 µL of **FILTERED** 5 mM MOPS. Mix the contents of the vial using the vortex mixer for 10 seconds.
- Place beakers in the aspirator to collect the wash.
- Prepare each cartridge by running 5 mL of methanol followed by 5 mL of deionized water. Make sure the column packing does not dry out.
- Load sample onto the cartridge. Wash the sample through the cartridge and with 2 mL of deionized water. This fraction contains mostly salts and should be discarded.
- Place the vial holder in the aspirator. Place the appropriate vials in position to collect eluted steroids.
- Apply 0.5 mL of 100 % methanol. Collect the entire eluate.
- Dry the sample in the speed vac. This step takes about 60 minutes.

D) Prepare sample for capillary electrophoresis

- Reconstitute sample in 30 µL of Buffer 1 (i.e. stacking buffer)
- Mix the contents of the vial using the vortex mixer for 1 minute. Spin the vial using the bench top picofuge for 10 seconds. Cover the vial with parafilm, and sonicate it for 1 minute. Remove parafilm. Spin the vial for 10 seconds with a picofuge.
- Transfer sample into a labeled PCR vial (catalog #20170-012, VWR). Spin the PCR vial and degas it for 1 minute in the bench top vacuum degasser.
- Spin the vial again if bubbles are visible.
- Run samples on the capillary electrophoresis instrument

Table S1 Comparison of Steroid Stacking with Different Concentrations of CAPS

	17α,20β-dihydroxy-pregn-4-en-3-one	testosterone	11-keto testosterone	estrone	17β-estradiol	17α-ethinyl estradiol
[1]Peak Area Obtained with 5 mM CAPS						
Day 1	404 ± 7	690 ± 30	260 ± 20	1210 ± 40	2760 ± 50	3200 ± 100
Day 2	470 ± 40	590 ± 10	226 ± 9	830 ± 40	1930 ± 60	2160 ± 30
Day 3	560 ± 10	560 ± 30	260 ± 10	740 ± 10	2170 ± 70	2120 ± 80
[2]Peak Area Obtained with 50 mM CAPS						
Day 1	76 ± 3	140 ± 10	60 ± 2	310 ± 10	550 ± 30	611 ± 9
Day 2	81 ± 5	100. ± 10	56 ± 6	220 ± 20	350 ± 10	430 ± 40
Day 3	97 ± 5	100. ± 10	69 ± 6	210 ± 10	418 ± 7	380 ± 10
[3]Observed Enhancement in Peak Area						
Day 1	5.3 ± 0.2	4.9 ± 0.4	4.3 ± 0.4	3.9 ± 0.2	5.0 ± 0.3	5.2 ± 0.2
Day 2	5.8 ± 0.6	5.9 ± 0.6	4.0 ± 0.5	3.8 ± 0.4	5.5 ± 0.2	5.0 ± 0.5
Day 3	5.8 ± 0.3	5.6 ± 0.6	3.8 ± 0.4	3.5 ± 0.2	5.2 ± 0.2	5.6 ± 0.3
Average	5.6 ± 0.7	5.5 ± 0.9_7	4.0 ± 0.7	3.7 ± 0.5	5.3 ± 0.4	5.3 ± 0.6

[1]Conductivity (σ) of 5 mM CAPS is estimated at 1.2 x 10^{-5} $m^2CV^{-1}s^{-1}$, when calculated according to the equation: $\sigma = F\Sigma C_i\mu_i z_i$, where F is the Faraday constant, C_i is the concentration, z_i is charge of the ion and μ_i is the electrophoretic mobility of the ion. The conductivity calculation was based on all the ion species present in solution for each buffer.

[2]Conductivity (σ) of 50 mM CAPS is estimated at 9.5 x 10^{-5} $m^2CV^{-1}s^{-1}$, when calculated according to the equation: $\sigma = F\Sigma C_i\mu_i z_i$, where F is the Faraday constant , C_i is the concentration, z_i is charge of the ion and μ_i is the electrophoretic mobility of the ion. The conductivity calculation was based on all the ion species present in solution for each buffer.

[3]Enhancement in peak area is calculated as the area ratio (5 mM/50mM CAPS) obtained each day.

Table S2 Replicate Measurements of Plasma Spiked with 1.5 Picomoles (50 nM) Steroids

	17α,20β-dihydroxy-pregn-4-en-3-one	testosterone	11-keto testosterone	estrone	17β-estradiol	17α-ethinyl estradiol
Injection 1	49 ± 1	44 ± 1	51 ± 1	51 ± 2	49 ± 3	49 ± 2
Injection 2	48 ± 1	39 ± 1	48 ± 1	47 ± 2	49 ± 3	48 ± 2
Injection 3	46 ± 1	40 ± 1	47 ± 1	52 ± 2	43 ± 3	46 ± 2

The peak corresponding to estrone obtained following the third injection was split into two peaks. The area of both peaks were measured and the sum of the areas used to calculate the concentration of estrone from the calibration curve.

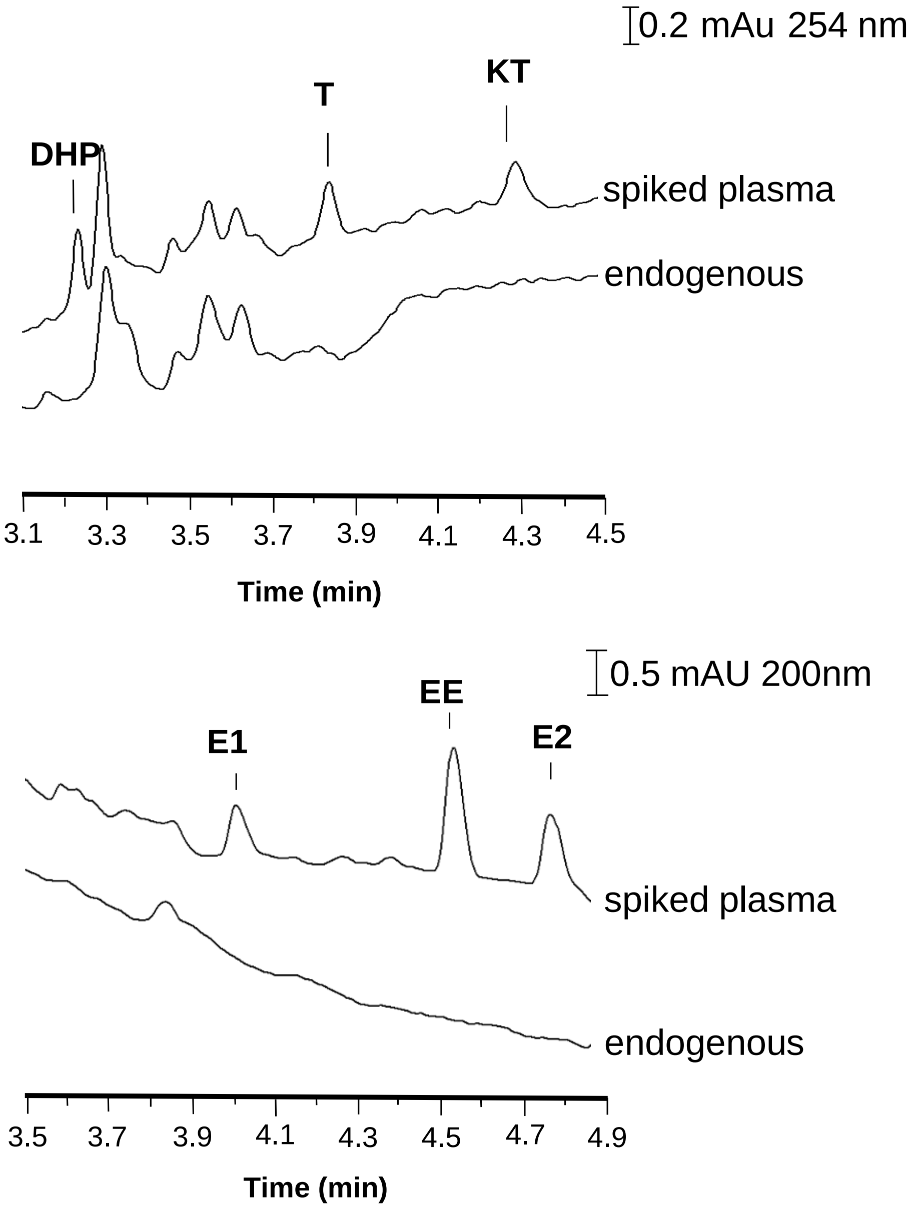

Fig. S1A Electropherogram used to determine steroid recovery from plasma pooled from two female fish that is split into two fractions. One fraction of plasma is spiked with 1.5 picomoles of each steroid, which when reconstituted and quantified generates a concentration of 50 nM, when 100% recovery is achieved. The second fraction is measured to assess the endogenous level of circulating steroids. As the fish are reproductively in active, no circulating steroids are present in the endogenous trace. Blood is transferred to a centrifuge tube containing 2 μL of aqueous heparin (6.5. mg/ mL deionized water) and then processed as described in this supporting information. Separation conditions are described in the text. Peak labels and heights are as follows: estrone (E1) height 568, 17α-ethinyl estradiol (EE) height 1233, 17-β estradiol (E2) height 855, 17α,20β-dihydroxy-pregn-4-en-3-one (DHP) height 392, testosterone (T) height 283, and 11-ketotestosterone (KT) height 216

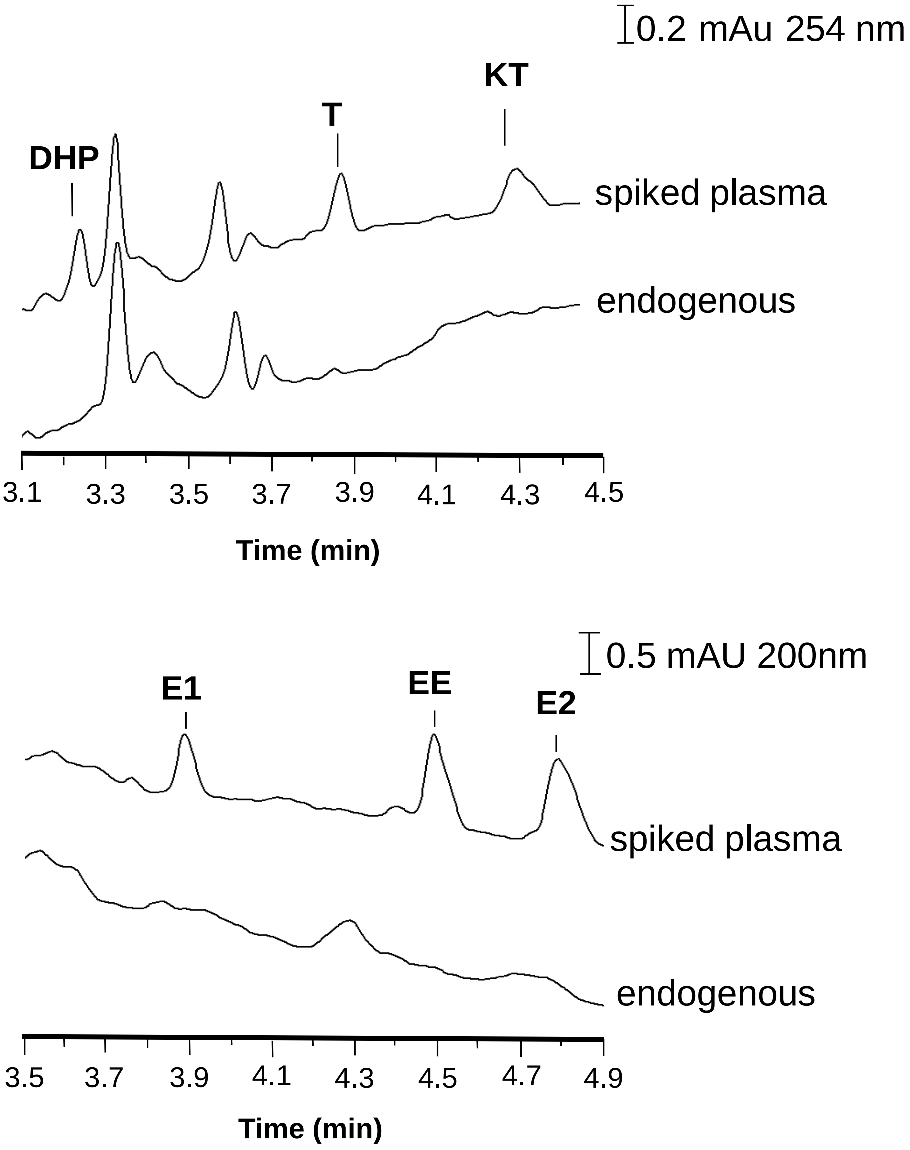

Fig. S1B Electropherogram used to determine steroid recovery from plasma pooled from two female fish that is split into two fractions. One fraction of plasma is spiked with 1.5 picomoles of each steroid, which when reconstituted and quantified generates a concentration of 50 nM, when 100% recovery is achieved. The second fraction is measured to assess the endogenous level of circulating steroids. As the fish are reproductively in active, no circulating steroids are present in the endogenous trace. Blood is transferred to a centrifuge tube containing 2 µL of deionized water and then processed as described in this supporting information. Separation conditions are described in the text. Peak labels and heights are as follows: estrone (E1) height 585, 17α-ethinyl estradiol (EE) height 907, 17-β estradiol (E2) height 779, 17α,20β-dihydroxy-pregn-4-en-3-one (DHP) height 353, testosterone (T) height 333, and 11-ketotestosterone (KT) height 230

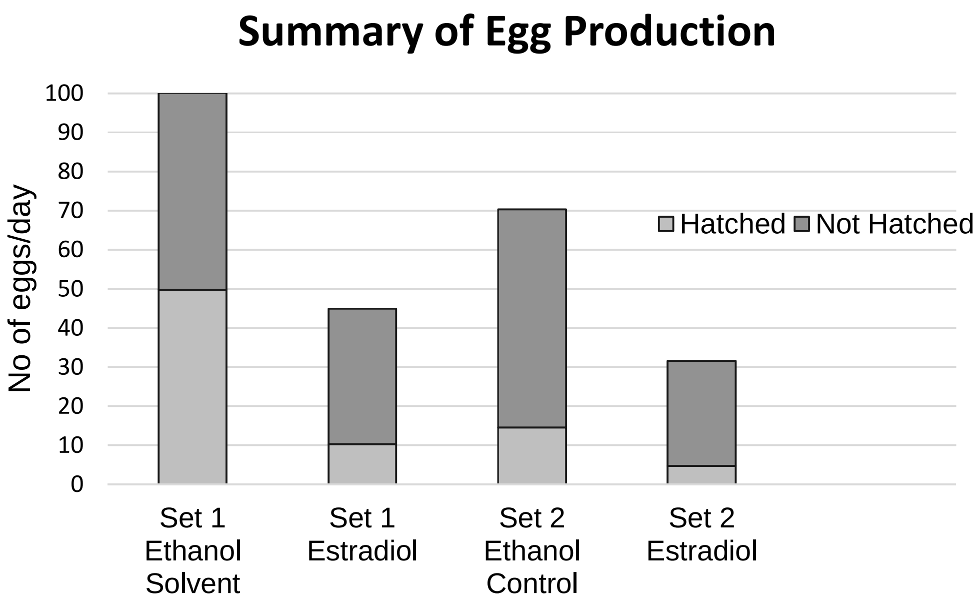

Fig. S2 Summarizes the egg production and viability for set 1 fish (16.9 weeks of age) and set 2 fish (33.3 weeks of age)
